# Species composition, seasonal abundance and distribution of potential anopheline vectors in a malaria endemic area of Iran: field assessment for malaria elimination

**DOI:** 10.1186/s12936-019-2795-x

**Published:** 2019-05-02

**Authors:** Alireza Sanei-Dehkordi, Moussa Soleimani-Ahmadi, Seyed Aghil Jaberhashemi, Mehdi Zare

**Affiliations:** 1 Social Determinants in Health Promotion Research Center, Hormozgan University of Medical Sciences, Bandar Abbas, Iran; 2 Department of Medical Entomology and Vector Control, Faculty of Health, Hormozgan University of Medical Sciences, P.O. Box: 79145-3838 Bandar Abbas, Iran; 3 Bashagard Health Center, Hormozgan University of Medical Sciences, Bashagard, Iran; 4 Department of Occupational Health Engineering, Faculty of Health, Hormozgan University of Medical Sciences, Bandar Abbas, Iran

**Keywords:** *Anopheles*, Malaria, Topography, Bashagard, Iran

## Abstract

**Background:**

Despite decreases in incidence, malaria remains a major public health challenge in south and southeast Iran, where vector control is considered one of the main strategies for elimination of the disease. The efficacy of this strategy depends on understanding malaria vector ecology, which varies by species. This study was conducted to determine the species composition, seasonal abundance and distribution of potential anopheline vectors in Bashagard County, one of the important malaria-endemic areas in south Iran.

**Methods:**

In this cross-sectional study, four typical foothill and mountainous villages were selected by simple random sampling. Anopheline mosquitoes were collected by the standard dipping method for larvae and total catch for adults. Anopheline specimens were morphologically identified using taxonomic keys. Statistical analyses were performed using SPSS ver.20 software.

**Results:**

In total, 1211 anopheline specimens, including 1055 (87.12%) larvae and 156 (12.88%) adults, were collected and identified. They consisted of 9 species: *Anopheles moghulensis* (27.89%), *Anopheles dthali* (18.91%), *Anopheles culicifacies* (16.60%), *Anopheles stephensi* (15.38%), *Anopheles turkhudi* (9.83%), *Anopheles superpictus* (9.66%), *Anopheles apoci* (1.40%), *Anopheles fluviatilis* (0.17%), and *Anopheles sergentii* (0.08%). The most prevalent species in adult catches were *An. culicifacies* (44.23%), *An. dthali* (21.15%) and *An. stephensi* (16.03%), and the most prevalent species caught as larvae were *An. moghulensis* (31.94%), *An. dthali* (18.85%) and *An. stephensi* (15.26%). Most of the anopheline species were distributed in different topographical areas and two proven malaria vectors, *An. culicifacies* and *An. superpictus,* were significantly associated with altitude and collected more frequently in the foothill regions. Most of the anopheline species were present almost throughout the year with a major peak in April and a smaller peak in October.

**Conclusion:**

The results of this study revealed that there are five malaria vectors in Bashagard County and some of them are more abundant in areas with special topographic features and are reproductively active throughout the year. These findings can be applied to successful planning and providing effective control measures in problematic areas during the malaria elimination phase in Iran.

## Background

Mosquitoes have an important role in the transmission and disease dynamics of pathogens such as malaria, lymphatic filariasis, and arboviruses [1]. Malaria is considered a serious mosquito-borne disease, with a great influence on human health caused by parasites of the genus *Plasmodium*, and is transmitted by female *Anopheles* mosquitoes [2]. According to WHO estimation, there were 219 million cases and 435,000 deaths from malaria in 2017 worldwide [3]. It is also one of the most important mosquito-borne diseases in Iran, especially along the international border with Pakistan [4]. Bashagard is one of the malaria foci in this area and indigenous malaria cases are reported from different parts of this county [5]. The region experiences active malaria circulation, especially during the peak of malaria vector breeding seasons, mostly in the spring and autumn [4].

A malaria eradication programme was initiated in 1951 in Iran and changed to malaria control in 1985 because of challenges and restrictions [6]. Iran has been in the current elimination stage since 2010. In 2009, the number of malaria cases was 6122, but reduced to 939 in 2017 [3, 7]. Despite years of dominant malaria vector control plans, sporadic outbreaks occur in the malarious areas, including Bashagard County in southeast Iran. In this county during 2008–2018, 1411 cases of malaria were reported, out of which *Plasmodium vivax* and *Plasmodium falciparum* accounted for 1399 (99.15%) and 12 (0.85%) cases, respectively (Bashagard Health Centre, unpublished data, 2018).

In an elimination phase, vector control strategy is focused on reduction of the vectorial capacity of the vector populations below a critical level needed to maintain transmission [8]. This strategy is also the main approach to reduce malaria transmission in the endemic areas in many parts of the world and it is considered the most effective measure for malaria eradication [9].

A full understanding of environmental features such as altitude, topography, meteorological conditions, climate, and water body characteristics which affect development, abundance and seasonal activity of malaria vectors can be considered an important factor in malaria control programmes [10, 11]. Therefore, it is critical to understand the relationship between distribution of anopheline mosquitoes and environmental parameters in different areas prior to the implementation of any vector control programme [10].

There are eight malaria vectors in Iran: *Anopheles culicifacies* sensu lato (s.l.), *Anopheles dthali*, *Anopheles fluviatilis* s.l., *Anopheles maculipennis* s.l., *Anopheles sacharovi*, *Anopheles stephensi* and *Anopheles superpictus* are proven vectors, while *Anopheles pulcherrimus* is reported as a suspected vector [5]. All the malaria vectors of the country except *An. maculipennis* s.l. and *An. sacharovi* are found in Hormozgan Province [5, 12]. In Bashagard County, five malaria vectors: *An. stephensi*, *An. fluviatilis* s.l., *An. dthali*, *An. culicifacies* s.l., and *An. superpictus* s.l. have been reported previously, where *An. stephensi* is a primary vector and other species are secondary vectors [12, 13].

Since the malaria control programme in Iran has focused on targeting malaria vectors through indoor residual spraying (IRS) of insecticide, long-lasting insecticidal nets (LLINs), and use of larvicides, there is a need for a full understanding of the bionomics of vectors to interrupt malaria transmission in endemic areas such as Bashagard County. Moreover, to support vector control programmes, information on distribution and speciation of malaria vectors is important to determine what type of elimination measures are most appropriate [14]. This study was conducted in Basagard County to determine species composition, seasonal abundance and distribution of anopheline mosquitoes. The results of this study will provide useful information to help in designing and implementing effective programmes for vector control in the study area during the elimination phase of the National Malaria Control Programme.

## Methods

### Study area

**Fig. 1:**
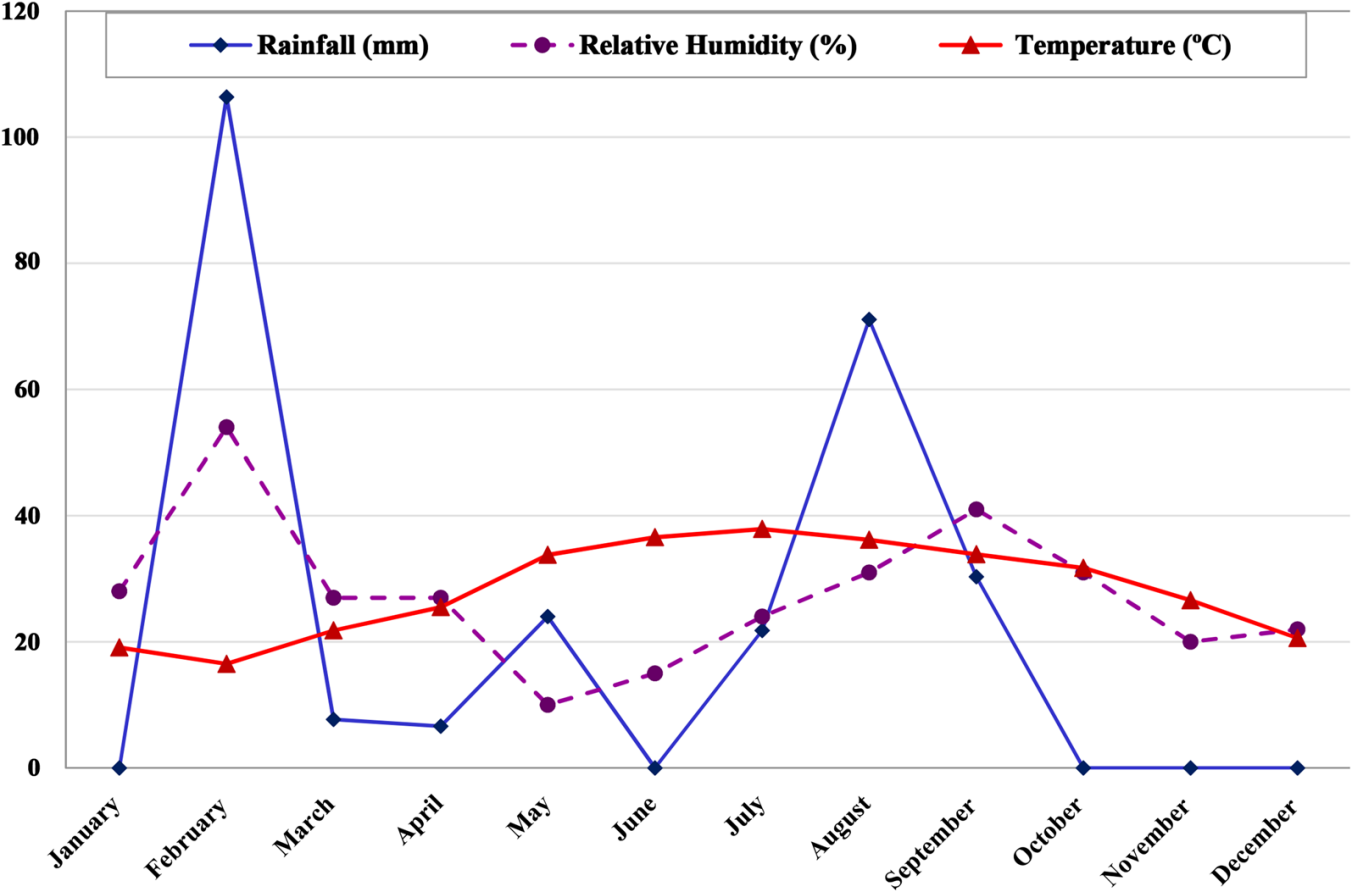
Average of meteorological parameters during 2016–2017 in Bashagard County, south of Iran

The study was carried out in Bashagard County, Hormozgan Province, southeast Iran. This county is largely rural, with an approximate population of 43,000 in 2016 and a 16,000 km^2^ area located between latitude 26°04′–26°58′ N and longitude 57°23′–59°02′ E. In this county, the average annual rainfall is 251 mm and the annual averages of minimum and maximum relative humidity are respectively 10% in May and 54% in February. The climate in this region is warm with a mean annual temperature of 28.3 °C, ranging from 19.1 to 37.9 °C (Fig. 1).

The county is mostly hilly with population inhabited mostly close to water bodies such as streams and rivers. The villages are small with low number of population who live in shelters made of palm tree branches and houses made of cement blocks. Livestock herding and agriculture are the main occupations in this area.

### Study design

**Fig. 2:**
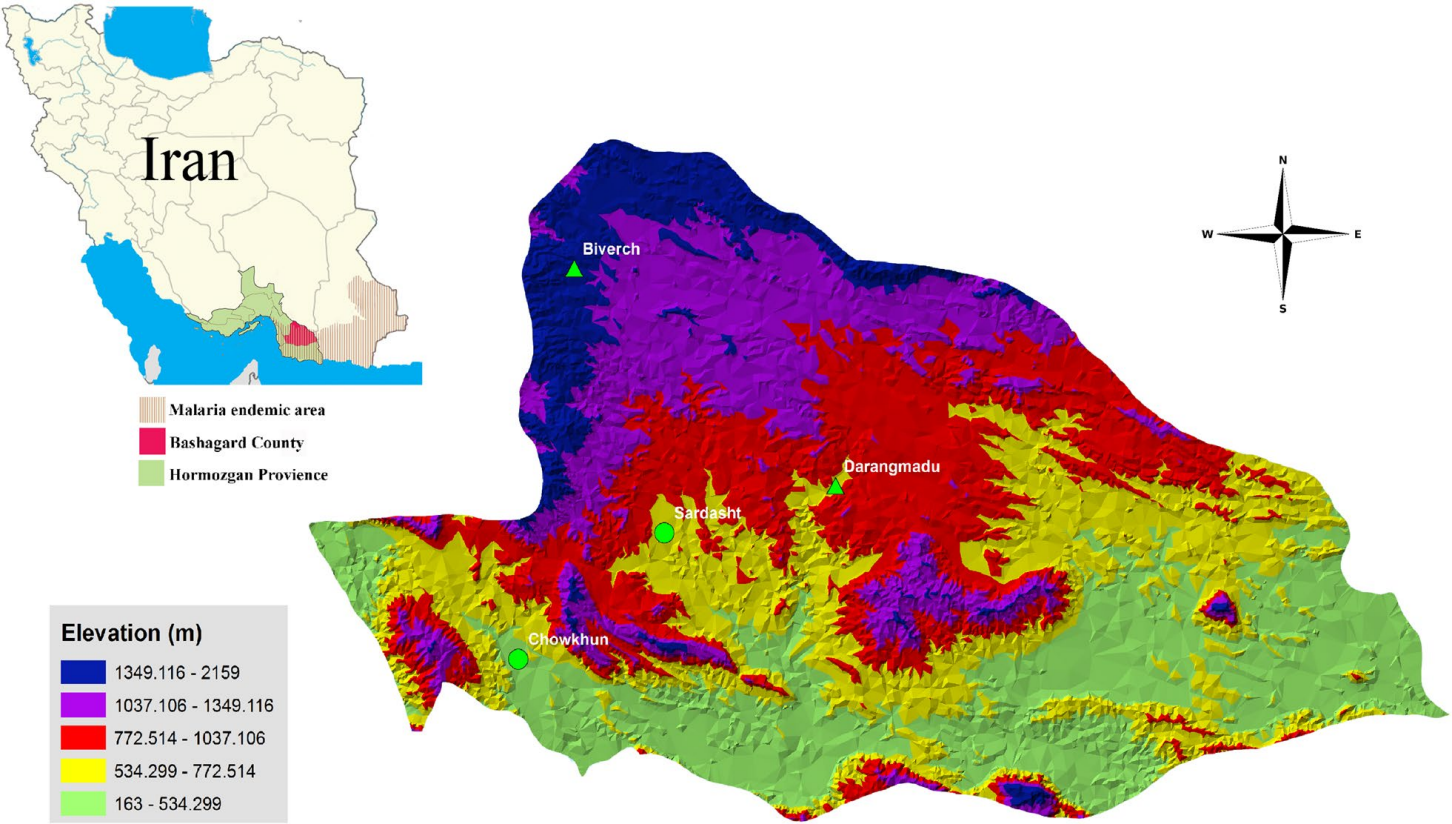
Map showing the provinces of Iran, highlighting the location of malaria-endemic areas and study villages in Bashagard County of Hormozgan Province

Based on the reported malaria cases during past years, human population densities and suitability of places for mosquito collection, four villages with different topographical characteristics in the study area were selected by simple random sampling. Anopheline mosquitoes were collected from selected villages: Sardasht (26°27′N, 57°54′E, 710 m) and Chowkhun (26°18′N, 57°41′E, 438 m) in foothill area, and Biverch (26°47′N, 57°47′E, 1382 m) and Darangmadu (26°30′N, 58°09′E, 745 m) in a mountainous area (Fig. 2).

### Mosquito collections and identification

A monthly entomological study was conducted from September 2016 to August 2017 in four villages of Bashagard County. Anopheline larvae were collected from oviposition sites in and within a 500-m radius of each village by a standard 350-ml mosquito dipper according to WHO procedures [15]. Where mosquito larvae were found, depending on the size of each larval breeding place, 10–30 dips were taken. In the small habitats, where dippers were not effective, plastic pipettes were used for larval collection. Sampling was always performed by the same individual for about 30 min at each larval breeding place and all third and fourth instar larvae were preserved in the lacto-phenol. In the laboratory, each specimen was mounted in Berlese’s medium on a microscope slide and identified to species using the morphological characters [16].

To collect female anopheline mosquitoes in each village, four houses were selected as fixed sampling sites and four houses were selected randomly as variable sampling sites. Anopheline mosquitoes were collected monthly by total catch (spray sheet collection) in the selected houses early in the morning. The surfaces in the spray catch rooms were covered using white sheets, and the rooms were sprayed with pyrethrum, and after 10 min the knocked-down mosquitoes were collected. In addition, adult anopheline mosquitoes resting indoors were collected using an aspirator and a flashlight.

Mosquitoes were collected at all stations during the study period and brought to the field laboratory for processing and identification. Finally, *Anopheles* species were morphologically identified using taxonomic key of Iranian mosquitoes [16].

### Statistical analysis

The data were analysed using SPSS Ver. 20. Chi squared test was used to determine the relationship between topographic type and mosquito density. All statistical analyses were performed at 5% significance.

## Results

### *Anopheles* species composition and abundance

**Fig. 3:**
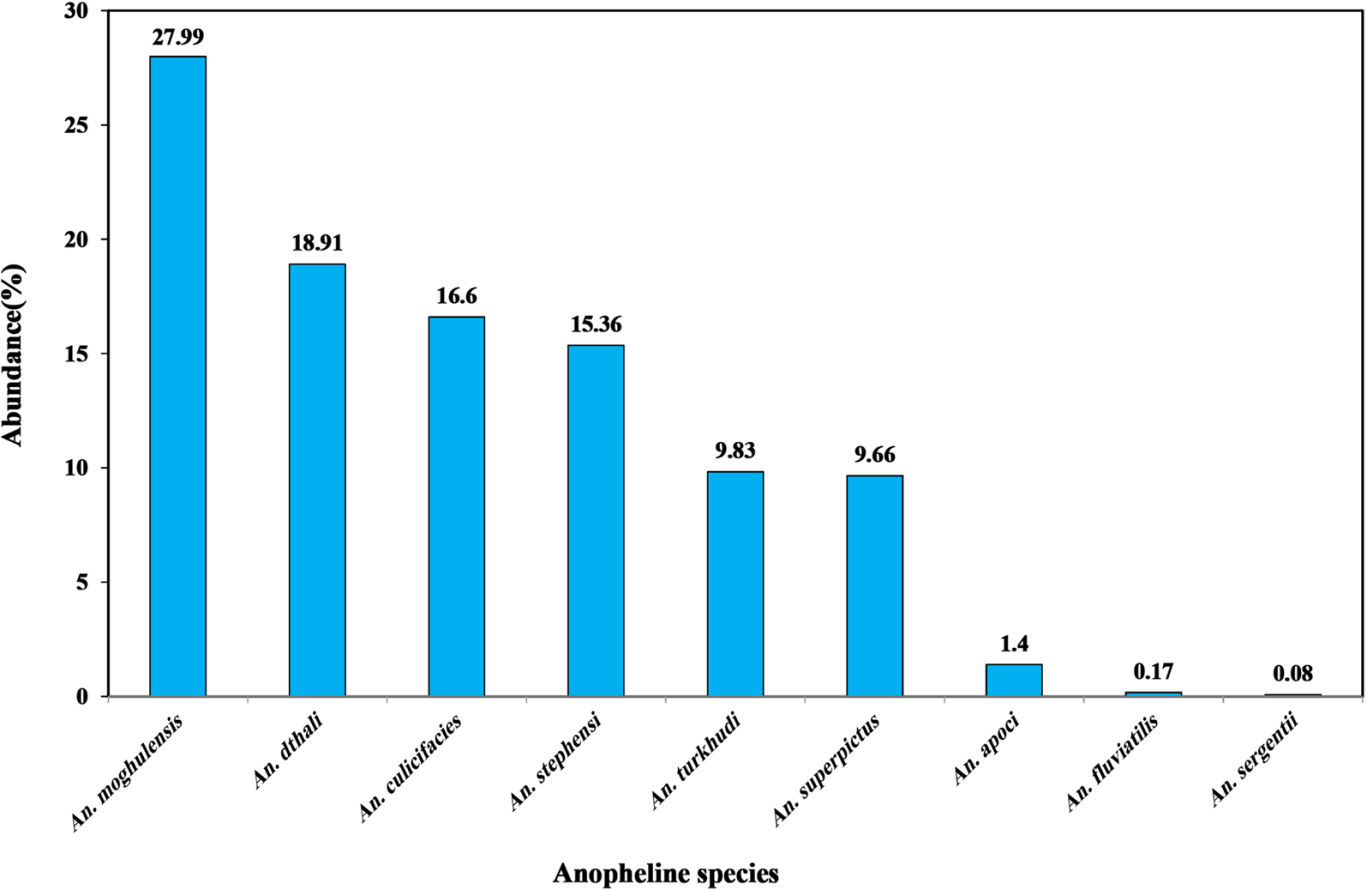
Abundance of anopheline species in Bashagard County, south of Iran

In this study, 1211 anopheline specimens including 1055 (87.12%) larvae and 156 (12.88%) adults, belonging to nine species were collected by different methods. A summary of anopheline species composition and abundance is shown in Fig. 3. A total of 1055 anopheline larvae, representing seven species were collected and identified (Table 1). The most abundant anopheline species were *An. moghulensis* (31.94%), *An. dthali* (18.85%) and *An. stephensi* (15.26%), which together accounted for 66.05% of the total anopheline larvae collected. The least collected *Anopheles* were *An. culicifacies* s.l. (12.51%), *An. turkhudi* (11.00%), *An. superpictus* (9.10%), and *An. apoci* (1.60%).

**Table 1:** Abundance of the anopheline larvae in different topographical areas of Bashagard County, south of Iran

Species	Topographic areas		
	Foothill	Mountainous	Total
	No. (%)	No. (%)	No. (%)
*An. moghulensis*	209 (33.82)	128 (29.29)	337 (31.94)
*An. dthali*	122 (19.74)	74 (16.93)	196 (18.58)
*An. culicifacies*	59 (9.55)	73 (16.70)	132 (12.51)
*An. stephensi*	82 (13.27)	79 (18.08)	161 (15.26)
*An. turkhudi*	84 (13.59)	32 (7.32)	116 (11.00)
*An. superpictus*	48 (7.77)	48 (10.98)	96 (9.10)
*An. apoci*	14 (2.27)	3 (0.69)	17 (1.61)
Total	618 (100)	437 (100)	1055 (100)

During this study, eight female anopheline species were collected from houses in the studied locations (Table 2). *Anopheles culicifacies* (44.23%), *An. dthali* (21.15%)*, An. stephensi* (16.03%), and *An. superpictus* (13.44%) were the predominant species and *An. turkhudi* (1.92%), *An. fluviatilis* (1.28%), *An. moghulensis* (1.28%), and *An. sergentii* (0.64%) were generally the least abundant in the study area. In this study. *An. apoci* was collected only in larval stage and *An. fluviatilis* and *An. sergentii* were collected only in adult stage in houses.

**Table 2:** Abundance of the adult anopheline mosquitoes in different topographical areas of Bashagard County, south of Iran

Species	Topographic areas		
	Foothill	Mountainous	Total
	No. (%)	No. (%)	No. (%)
*An. moghulensis*	2 (1.94)	0	2 (1.28)
*An. dthali*	17 (16.50)	16 (30.19)	33 (21.15)
*An. culicifacies*	45 (43.69)	24 (45.28)	69 (44.23)
*An. stephensi*	16 (15.53)	9 (16.98)	25 (16.03)
*An. turkhudi*	3 (2.91)	0	3 (1.92)
*An. superpictus*	17 (16.53)	4 (7.55)	21 (13.46)
*An. fluviatilis*	2 (1.94)	0	2 (1.28)
*An. sergentii*	1 (0.97)	0	1 (0.64)
Total	103 (100)	53 (100)	156 (100)

### Abundance of the anopheline mosquitoes in different topographical areas

In the present study, 1055 larval specimens were collected from natural larval habitats in different topographical areas. Out of these, 618 (58.6%) and 437 (41.4%) were collected from the foothill and mountainous areas, respectively. There was a significant difference in larval abundance in these areas (*P* < 0.05).

A total of 156 females *Anopheles*, was collected from foothill (n = 103, 66%) and mountainous (n = 53, 34%) areas. *Anopheles* abundance in the foothill was significantly higher than in the mountainous areas (*P *= 0.023). *Anopheles dthali* was the most abundant malaria vector, accounting for 19.39 and 18.37% of all recorded anophelines in foothill and mountainous areas, respectively, and 18.91% of the total sample from both areas combined. According to the statistical analysis, there was no significant association between abundance of *An. dthali* and topographic type in the study areas (*P *= 0.243). *Anopheles culicifacies* s.l. was collected from various localities in the study areas and represented 25.4 and 20.4% of the total anopheline samples in foothill and mountainous areas, respectively, and 16.60% of the total in both areas. There was a significant difference in the distribution of *An. culicifacies* in foothill and mountainous areas (*P *= 0.005).

*Anopheles stephensi* was found abundantly with varying densities in all of the study areas. This *Anopheles* was also more common in foothill areas and densities of female mosquitoes were generally higher in the moderately low altitude localities. Chi squared analyses showed no significant relationship between prevalence of *An. stephensi* and topographic type in the study areas (*P *= 0.223).

*Anopheles superpictus* was collected from various localities in the studied areas, but its adult stage was more prevalent in foothill areas. Statistical analysis showed that there was a significant relationship between abundance of *An. superpictus* adult female and altitude of the study areas (*P *= 0.009).

*Anopheles fluviatilis* s.l. was collected in low numbers and only 2 specimens were caught during total catches from houses in the foothill region (Table 2).

A non-vector species, *An. moghulensis*, was the most prevalent species in the study areas and a high proportion of the total collection of larvae (63.11%) were *An. moghulensis*. A significant relationship was found between the abundance of this species and topographic type in the study areas (*P *= 0.042).

Another non-vector species, *An. turkhudi*, was more prevalent in foothill regions. Increasing the altitude was a significant factor affecting the distribution and abundance of *An. turkhud* in the studied areas (*P *= 0.044).

*Anopheles apoci*, the third found non-vector anopheline species, was collected with a low larval number in the study areas (Table 1). There was no significant association between abundance of *An. apoci* larvae in foothill and mountainous regions (*P *= 0.228).

*Anopheles segentii*, the fourth non-vector species, was the least common species and only one adult specimen was collected from the foothill regions (Table 2).

### Seasonal abundance of *Anopheles* species

**Fig. 4:**
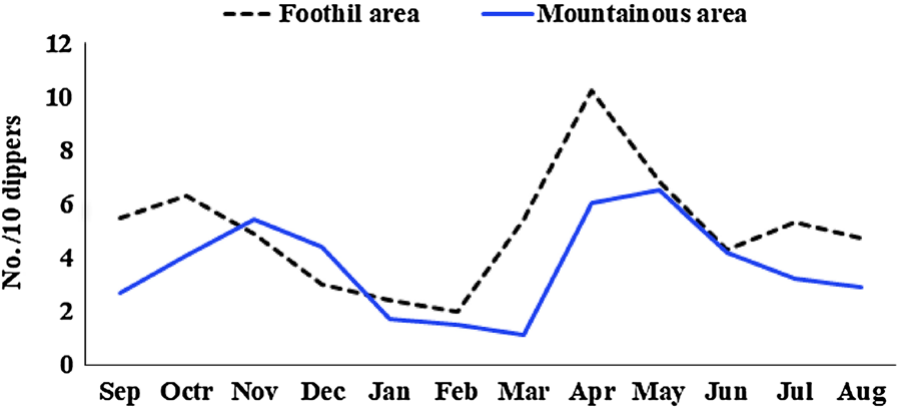
The monthly mean density of anopheline larvae in different topographical areas of Bashagard County, south of Iran

The results of the monthly larval collection showed that most of the anopheline species were present almost throughout the year. In mountainous regions, larval activity reached a major peak in April and a smaller peak in October while in foothill regions two peaks of larval activity occurred during November and May (Fig. 4).

The monthly activity of female mosquitoes in human resting places revealed similar population dynamics in mountainous and foothill regions. In both regions, mosquitoes remained reproductively active throughout 10 months of the year, starting in February, reaching a major peak in April and then decreasing gradually and after that, the activity increases and reaches the second peak in October.

**Fig. 5:**
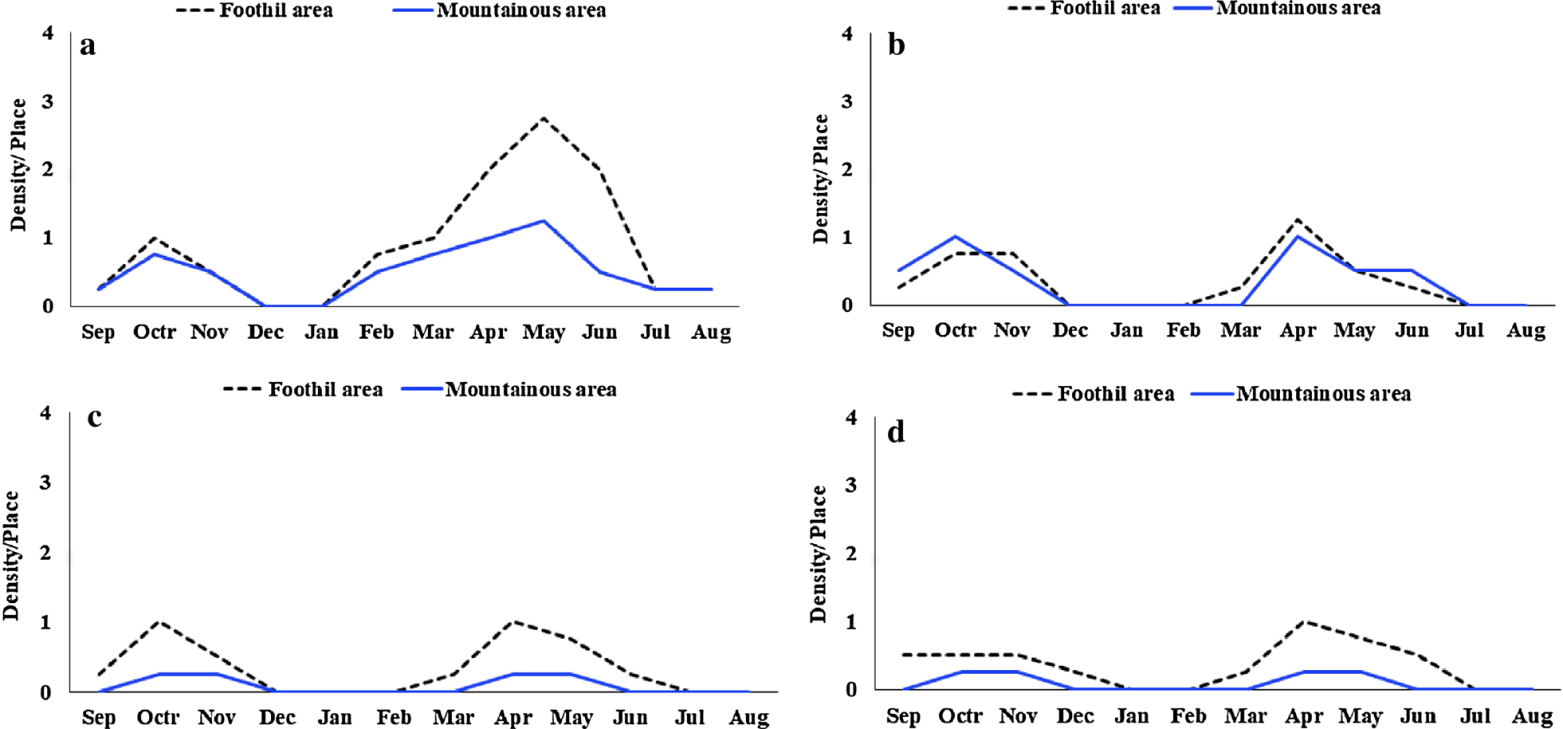
The monthly mean indoor-resting density of malaria vectors in different topographical areas of Bashagard County, south of Iran. *An. culicifacies* (**a**), *An. dthali* (**b**), *An. stephensi* (**c**) and *An. superpictus* (**d**)

The seasonal distribution of the most abundant species in human places revealed population fluctuations in different months. *Anopheles culicifacies* and *An. dthali* were the dominant species present almost throughout the year except cold months (December-February), with major peaks in April and smaller peaks in October. *Anopheles stephensi* and *An. superpictus* were the second-most frequently collected species throughout the sampling period with two peaks in April and October. The population of these species decreased greatly in July and September, and they were not found during December and February (Fig. 5).

Non-vector species, including *An. turkhudi*, *An. moghulensis* and *An. sergentii,* were collected in small numbers only in foothill regions during April and May.

## Discussion

In spite of the prominent success in malaria control in the past decades, malaria remains prevalent in vulnerable areas, especially in the south and southeast of Iran, where suitable climatic conditions have resulted in the distribution of several malaria vector species. This study provided baseline information for the species composition, seasonal abundance and distribution of potential malaria vectors in Bashagard County, an area with seasonal malaria transmission in southern Iran. The results revealed the occurrence of nine different *Anopheles* species in Bashagard County, which included five out of the eight proven malaria vectors in Iran and this may be associated with malaria transmission dynamics in this county. These species included *An. stephensi*, which is known as the primary vector, and *An. dthali*, *An. culicifacies*, *An. fluviatilis,* and *An. superpictus,* which are considered secondary vectors in the south and southeast of the country [12, 17]. The richness of *Anopheles* species in this county may be explained by the existence of different mosquito oviposition sites, which are close to the river and human habitats which may provide more food sources and ecological niches for anopheline species [18].

During the study, anopheline mosquitoes were frequently collected from foothill areas. This could be due to different ecological factors. One could be abundant mosquito breeding places such as rivers in this area that often have low levels of water, which creates favourable conditions for mosquito breeding. Another reason could be the existence of valleys along river beds, which can serve as resting sites for adult mosquitoes. Moreover, in foothill areas villages are usually placed close to the rivers, providing blood sources and resulting in high population density of mosquitoes. It has previously been reported that in foothill areas in the south of Iran, malaria is often transmitted by vectors exhibiting a very high degree of exophily, which is a challenge for malaria elimination in this area [19].

This study showed that *An*. *dthali*, *An. culicifacies* and *An. stephensi* were the most widespread malaria vectors and collected from all topographic areas almost throughout the year. The higher peaks of the larvae and adult species density were observed during April and the smaller peaks during October, after two rainy seasons that create favourable breeding places for anophelines. This implies that these months can be malaria threat times in the study area, which is after one of the two malaria transmission seasons in Bashagard that occurs from April to June and October to December [4].

In this study, *An*. *dthali* was frequently collected from foothill areas. Similar abundance has been reported for this *Anopheles* from Minab, the neighbouring district to Bashagard [20]. Comparison of the findings with those of other studies confirms the distribution of *An*. *dthali* in southern regions of the Zagros Mountains and coastal area of the Persian Gulf up to 1410 m [12, 21]*. Anopheles dthali* has been reported as a secondary vector in southern Iran and in northern areas of Saudi Arabia and Somalia [12]. In southern parts of Iran, sporozoite rates for this species has been reported to be 0.67–2.08% [21]. Therefore, although this *Anopheles* contributes to malaria transmission in Bashagard County, it is unlikely that it could have a substantial impact on malaria transmission in the absence of the major vectors in this county.

The results indicated that *An. culicifacies* was a common species in the study area and collected from all the topographic regions. This species has mainly been reported from Sistan and Baluchistan, Kerman and Hormozgan Provinces in southeastern Iran [22]. Among the secondary malaria vectors in Iran, *An. culicifacies* can be considered a potential malaria vector, since its role in transmission has been reported from Sistan and Baluchestan Provinces in malaria-endemic areas bordering Afghanistan and Pakistan. For this anopheline species, sporozoite rate was reported 1–4.7% in the south and southeast of Iran [21]. During this study, *An. culicifacies* was mainly collected from foothill regions. This is in agreement with the findings in southeast Iran, where this species have been reported to be abundant in foothill areas with an altitude of up to 3000 m [21, 23].

According to the results, *An. stephensi* was prevalent in foothill regions and abundance of adult mosquitoes was generally higher in the moderately low altitude localities. Previous studies have shown that this species is the most prevalent anopheline in the malarious area of southern Iran at altitudes of up to 900 m [21]. Abundance of *An. stephensi* in Bashagard County may be due to its being adaptable to various larval habitats and surface water bodies, especially different seasonal and perennial rivers providing favourite conditions for its breeding. Moreover, high abundance of this species in the study area may be due to its tolerance to different climatic conditions, including humid and arid climates. This finding agrees with results from South Asia, including the Indian subcontinent and Pakistan and Arabian Peninsula, which have the same climatic conditions as that of the study area [22]. *Anopheles stephensi* is known as a malaria vector in Asia especially in Iran, Persian Gulf areas and India. The infection rate of this species in the south of Iran with *P. vivax* and mixed infection with *P. vivax* and *P. falciparum* has been reported to be 0.97 and 0.32%, respectively [21].

The study findings showed that *An. superpictus* was distributed in the studied region with varying densities in both foothills and mountainous regions. This finding is in agreement with a previous study in this area that revealed high distribution of *An. superpictus* in Bashagard County [12]. This species is the most distributed malaria vector in Iran and presents in both malaria-endemic and non-endemic areas of the country at altitudes of 50–2000 m across all climatic zones from arid to wet [21]. *Anopheles superpictus* is recognized as the major malaria vector in the central areas of Iran, and the secondary vector in the southern parts. In Iran, this *Anopheles* has been reported to be infected by sporozoite with an infection rate of 0.65–4.7 [20, 21]. It has a broad geographical distribution in Palearctic region, Middle Eastern countries, Afghanistan, Pakistan, India, northern Africa, and central and southern Europe, and has been known as a malaria vector in these regions [24].

In this study, *An. fluviatilis* was found only as adult and two females of this species were collected from houses in the foothill regions. In a previous study in this county, Soleimani-Ahmadi et al. collected this species with low frequency in night-biting catch on animal baits [12]. The low abundance of *An. fluviatilis* might be attributed to the geographical and environmental conditions of the county. This species mainly breeds in springs, pits around springs, rainfall pits, and coastal plains in the south of Iran [25]. Moreover, it has been reported that *An. fluviatilis* is considered a secondary vector with a sporozoite rate of 1.4–11% in foothill regions at altitudes of 50–1100 m along the foothills from south to southeast Iran [21].

In the present study, *An. moghulensis* in larval stage was the most dominant species and showed a wide range of distribution within the study areas. The same distribution and abundance has been reported in the study area and other parts in the southeast of Iran [12, 20, 25]. Despite the considerable number of larvae specimens collected, only two were females of this *Anopheles*. This may be due to its zoophilic and exophilic behaviour. This finding accords with earlier observations, which showed that *An. moghulensis* collected in outdoor habitats on animal baits [12]. This *Anopheles* is not incriminated to be a vector of malaria in Iran and its distribution is limited to the southeast of the country [26].

During the study, *An. turkhudi* was collected in relatively low numbers, more often in the foothill areas. It is evident that females of *An. turkhudi* avoid any contact with man or his dwellings as only three female mosquitoes were collected from houses in the foothill region during the study period. Previous findings also reported similarity in abundance of this species [12]. *Anopheles turkhudi* is recorded from the southern and some central areas of Iran and is not considered a malaria vector in the country [12, 26].

*Anopheles apoci* was captured only as larvae with low density in the study areas. This species is distributed in the south of Iran and has no role in malaria transmission in the country [20, 26].

*Anopheles sergentii* was the least abundant species in the study area and only one female specimen was collected from the foothill regions and attempts to collect larvae from the area were unsuccessful. This is the first record of *An. sergentii* in Bashagard County. The low number of this species could be due to its outdoor feeding and resting behaviour. This species is not recognized as a malaria vector in Iran and it is usually found in the southern and central areas of the country [26].

## Conclusions

This study revealed that anopheline species composition and distribution in Bashagard County varies slightly by geographical location, and mosquito population density fluctuates with seasonal weather dynamics. This study indicated the occurrence of five potential malaria vectors in the study areas and some of them are expected to be more abundant in areas with special topographic features and are reproductively active throughout the year. Appropriate interventions towards vector control should be designed for the control of both larval and adult mosquitoes, which may help in the suppression of vector density.

The presence and wide distribution of malaria vectors constitute a major potential health problem and may hamper efforts to eliminate malaria in Bashagar County, especially if the reservoir of *Plasmodium* parasites increases via immigration of infected persons from other endemic countries, such as Afghanistan and Pakistan. Understanding malaria vector ecology is essential to design effective strategies for sustaining malaria control and elimination, especially in southeast Iran. In this regard, the findings of this study can be considered for successful planning and providing effective control measures for problematic areas during the malaria elimination phase in Iran.

## Abbreviations

IRS: indoor residual spraying
LLINs: long-lasting insecticidal nets

## References

[1] Service M (2012). Medical entomology for students.

[2] Caminade C, Kovats S, Rocklov J, Tompkins AM, Morse AP, Colón-González FJ (2014). Impact of climate change on global malaria distribution. Proc Natl Acad Sci USA.

[3] WHO. World malaria report 2018. Geneva: World Health Organization; 2018. http://apps.who.int/iris/bitstream/handle/10665/275867/9789241565653-eng.pdf.

[4] Madani A, Soleimani-Ahmadi M, Davoodi SH, Sanei-Dehkordi A, Jaberhashemi SA, Zare M (2017). Household knowledge and practices concerning malaria and indoor residual spraying in an endemic area earmarked for malaria elimination in Iran. Parasit Vectors..

[5] Hanafi-Bojd AA, Vatandoost H, Oshaghi MA, Charrahy Z, Haghdoost AA, Zamani G (2012). Spatial analysis and mapping of malaria risk in an endemic area, south of Iran: a GIS based decision making for planning of control. Acta Trop.

[6] Edrissian G (2006). Malaria in Iran: past and present situation. Iran J Parasitol..

[7] Sheikhzadeh K, Haghdoost AA, Bahrampour A, Zolala F, Raeisi A (2016). Assessment of the impact of the malaria elimination programme on the burden of disease morbidity in endemic areas of Iran. Malar J..

[8] Karunamoorthi K (2011). Vector control: a cornerstone in the malaria elimination campaign. Clin Microbiol Infect.

[9] Raghavendra K, Barik TK, Reddy BP, Sharma P, Dash AP (2011). Malaria vector control: from past to future. Parasitol Res.

[10] Ndoen E, Wild C, Dale P, Sipe N, Dale M (2010). Relationships between anopheline mosquitoes and topography in West Timor and Java, Indonesia. Malar J..

[11] Ndenga B, Githeko A, Omukunda E, Munyekenye G, Atieli H, Wamai P (2006). Population dynamics of malaria vectors in western Kenya highlands. J Med Entomol.

[12] Soleimani-Ahmadi M, Vatandoost H, Shaeghi M, Raeisi A, Abedi F, Eshraghian MR (2012). Vector ecology and susceptibility in a malaria-endemic focus in southern Islamic Republic of Iran. East Mediterr Health J..

[13] Soleimani-Ahmadi M, Vatandoost H, Zare M (2014). Characterization of larval habitats for anopheline mosquitoes in a malarious area under elimination program in the southeast of Iran. Asian Pac J Trop Biomed..

[14] Bugoro H, Iro’ofa C, Mackenzie DO, Apairamo A, Hevalao W, Corcoran S (2011). Changes in vector species composition and current vector biology and behaviour will favour malaria elimination in Santa Isabel Province, Solomon Islands. Malar J..

[15] WHO (1975). Manual on practical entomology in malaria. Part II. Methods and techniques.

[16] Azari-Hamidian S, Harbach RE (2009). Keys to the adult females and fourth-instar larvae of the mosquitoes of Iran (Diptera: Culicidae). Zootaxa..

[17] Sanei-Dehkordi A, Soleimani-Ahmadi M, Akbarzadeh K, Salim Abadi Y, Paksa A, Gorouhi MA (2016). Chemical composition and mosquito larvicidal properties of essential oil from leaves of an Iranian indigenous plant Zhumeria majdae. J Essent Oil Bearing Plant..

[18] Hanafi-Bojd AA, Vatandoost H, Oshaghi MA, Haghdoost AA, Shahi M, Sedaghat MM (2012). Entomological and epidemiological attributes for malaria transmission and implementation of vector control in southern. Acta Trop.

[19] Schapira A, Zaim M, Raeisi A, Ranjbar M, Kolifarhood G, Nikpour F (2018). History of the successful struggle against malaria in the Islamic Republic of Iran.

[20] Soleimani-Ahmadi M, Vatandoost H, Zare M, Turki H, Alizadeh A (2015). Topographical distribution of anopheline mosquitoes in an area under elimination programme in the south of Iran. Malar J..

[21] Hanafi-Bojd AA, Azari-Hamidian S, Vatandoost H, Charrahy Z (2011). Spatio-temporal distribution of malaria vectors (Diptera: Culicidae) across different climatic zones of Iran. Asian Pac J Trop Med..

[22] Pakdad K, Hanafi-Bojd AA, Vatandoost H, Sedaghat MM, Raeisi A, Moghaddam AS (2017). Predicting the potential distribution of main malaria vectors *Anopheles stephensi*, *An. culicifacies s.l.* and *An. fluviatilis s.l.* in Iran based on maximum entropy model. Acta Trop.

[23] Vatandoost H, Emami SN, Oshaghi MA, Abai MR, Raeisi A, Piazzak N (2011). Ecology of malaria vector *Anopheles culicifacies* inmalarious area of Sistan va Baluchestan province, south-east Islamic Republic of Iran. East Mediterr Health J..

[24] Hanafi-Bojd AA, Sedaghat M, Vatandoost H, Azari-Hamidian S, Pakdad K (2018). Predicting environmentally suitable areas for *Anopheles superpictus* Grassi (s.l.), *Anopheles maculipennis* Meigen (s.l.) and *Anopheles sacharovi* Favre (Diptera: Culicidae) in Iran. Parasit Vectors..

[25] Hanafi-Bojd AA, Vatandoost H, Oshaghi MA, Charrahy Z, Haghdoost AA, Sedaghat MM (2012). Larval habitats and biodiversity of anopheline mosquitoes (Diptera: Culicidae) in a malarious area of southern Iran. J Vector Borne Dis..

[26] Sedaghat MM, Harbach RE (2005). An annotated checklist of the Anopheles mosquitoes (Diptera: Culicidae) in Iran. J Vector Ecol..

